# Effects of Transgenic *cry1Ca* Rice on the Development of *Xenopus laevis*

**DOI:** 10.1371/journal.pone.0145412

**Published:** 2015-12-22

**Authors:** Xiuping Chen, Jiamei Wang, Haojun Zhu, Yunhe Li, Jiatong Ding, Yufa Peng

**Affiliations:** 1 State Key Laboratory for Biology of Plant Diseases and Insect Pests, Institute of Plant Protection, Chinese Academy of Agricultural Sciences, Beijing 100193, China; 2 College of Animal Science and Technology, Yangzhou University, Yangzhou 225009, China; 3 Jiangsu Agri-animal Husbandry Vocational College, Taizhou 225300, China; Institut National de la Recherche Agronomique (INRA), FRANCE

## Abstract

In fields of genetically modified, insect-resistant rice expressing *Bacillus thuringiensis* (Bt) proteins, frogs are exposed to Bt Cry proteins by consuming both target and non-target insects, and through their highly permeable skin. In the present study, we assessed the potential risk posed by transgenic *cry1Ca* rice (T1C-19) on the development of a frog species by adding purified Cry1Ca protein or T1C-19 rice straw into the rearing water of *Xenopus laevis* tadpoles, and by feeding *X*. *laevis* froglets diets containing rice grains of T1C-19 or its non-transformed counterpart MH63. Our results showed that there were no significant differences among groups receiving 100 μg L^–1^ or 10 μg L^–1^ Cry1Ca and the blank control in terms of time to completed metamorphosis, survival rate, body weight, body length, organ weight and liver enzyme activity after being exposed to the Cry1Ca (*P* > 0.05). Although some detection indices in the rice straw groups were significantly different from those of the blank control group (*P* < 0.05), there was no significant difference between the T1C-19 and MH63 rice straw groups. Moreover, there were no significant differences in the mortality rate, body weight, daily weight gain, liver and fat body weight of the froglets between the T1C-19 and MH63 dietary groups after 90 days, and there were no abnormal pathological changes in the stomach, intestines, livers, spleens and gonads. Thus, we conclude that the planting of transgenic *cry1Ca* rice will not adversely affect frog development.

## Introduction

Transgenic rice expressing *Bacillus thuringiensis* (Bt) insecticidal proteins can effectively prevent and control lepidopteran pests, thus reducing the use of pesticides, but it has not yet been approved for commercial cultivation. A concern is that the planting of *Bt* rice may result in non-target effects [1]. Discussions about such effects have focused primarily on terrestrial organisms, such as non-target herbivorous insects [2,3], natural enemies [4,5], economically important insects [6,7], and soil organisms [8]. However, the potential effects of *Bt* crops on aquatic organisms has rarely been addressed, in particular since exposure of aquatic organisms to the plant-produced Cry proteins is regarded as being very low [9].

Studies have shown that the Bt toxins present in transgenic crop byproducts can enter stream ecosystems adjacent to agricultural fields through exudation from roots, dispersal of pollen and movement of post-harvest corn residues [9,10]. Douville et al. detected the existence of the *cry1Ab* gene in water, sediment and even in the tissues of mussels near *Bt* corn fields [11,12]. Tank et al. sampled the river water downstream of *Bt* corn fields and found that the soluble Cry1Ab concentration reached 21 ng L^–1^ [13]. Additionally, some studies have indicated potential adverse effects of *Bt* crops on aquatic organisms, including *Daphnia magna* [14–16], larvae of Trichoptera [17,18], larvae of a crane fly and an isopod [19]. Moreover, rice, unlike dry-land crops, requires water during most of its developmental stages. Wang et al. demonstrated that *Bt* rice releases detectable amounts of Bt protein into irrigation water [20]. Therefore, the risk of Bt rice for aquatic organisms needs to be addressed.

Frogs are commonly found in rice fields and play an important role in maintaining the biodiversity and stability of the paddy field ecosystem. However, frog populations have declined sharply worldwide in recent decades [21,22]. Frogs might be affected by *Bt* rice in three ways. First, frogs could ingest Bt proteins directly by consuming insects that have fed on *Bt* rice [23]. Second, because their skins are highly permeable, frogs could be exposed to Bt proteins that are released into the water [20]. Third, frog diets can be affected by changes in food resources. *Bt* rice effectively reduces the population of target insects, which may dramatically alter the composition of dominant insect species in a rice field as has for example been reported for cotton [24]. Therefore, it is important to assess the potential non-target effects of *Bt* rice on the development of frog species.

*Xenopus laevis* is a model animal widely used in environmental toxicology, because it is easy to feed, readily induced to lay eggs and very sensitive to external contamination [25]. In our previous study, we assessed the risk posed by transgenic rice expressing a Cry1Ab/1Ac fusion protein on the development of *X*. *laevis* froglets by a 90 day feeding test, and no adverse effects were observed [26]. Transgenic rice expressing *cry1Ca* (T1C-19) is a promising *Bt* rice line for commercial use that targets lepidopteran rice pests [27,28]. In the present study, *X*. *laevis* tadpoles were exposed to purified Bt Cry1Ab or *Bt* rice straw, and *X*. *laevis* froglets were fed diets containing rice grains of T1C-19 or its non-transformed isoline to assess the potential risk posed by *Bt* rice. Results from this study will provide important information concerning the environmental safety of *Bt* rice strains.

## Materials and Methods

### Ethics Statement

This study was approved by the Animal Research Committee of the Institute of Plant Protection, Chinese Academy of Agricultural Sciences. All procedures involving experimental animals were performed in accordance with the NIH guide for the Care and Use of Laboratory Animals. Briefly, all the animals were humanely treated during this study, the anesthetic procedure with 0.1% MS-222 (Sigma-Aldrich) was adopted, if necessary, to reduce the suffering of the experimental animals.

### Transgenic rice

T1C-19 rice expresses a gene encoding synthetic Cry1Ca under the control of the corn ubiquitin promoter and exhibits resistance to stem borers [27,28]. Its corresponding non-transformed isoline, MH63, is an elite *indica* restorer line for cytoplasmic male sterility and is commonly grown in China. Both rice lines were obtained from Huazhong Agricultural University (Wuhan, China). The two rice lines were simultaneously planted in two adjacent plots in the experimental field station of the Institute of Plant Protection, Chinese Academy of Agricultural Sciences (39.53°N, 116.70°E). Crops were cultivated according to commonly used local agricultural practices but without insecticide applications.

Rice was harvested at the end of October 2013, and rice stems of each line from 20 cm above the soil surface were collected and stored at −20°C until used. Conventional nutrient components of each rice line were analyzed by the Hangzhou Center for Inspection and Testing for Quality and Safety of Agricultural and Genetically Modified Products, Ministry of Agriculture, P. R. China.

### Purified Cry1Ca protein

Cry1Ca was purchased from Envirotest-China (agent for EnviroLogix Inc., Portland, Maine, USA; www.envirotest-china.com). The bioactivity of Cry1Ca was confirmed by performing sensitive insect bioassays in our laboratory using neonate *Chilo suppressalis* larvae fed an artificial diet containing a range of protein concentrations for 7 days. The dietary EC_50_ (toxin concentration resulting in 50% weight reduction compared to the control) was estimated to be 18.1 ng mL^–1^ [29].

### Animals

Mature female and male *X*. *laevis* were maintained separately in glass tanks containing dechlorinated water at 21 ± 2°C on a 12-h light/12-h dark cycle, and they were fed chopped pork liver once per week. One female/male pair of adult frogs was chosen and injected with 100 IU of human chorionic gonadotropin (Sigma-Aldrich, Saint Louis, MO, USA) to induce breeding. After eggs were laid, the female/male pair was removed from the breeding tank. Fertilized eggs were incubated at 22 ± 2°C on a 12-h light/12-h dark cycle. On the fifth day after emergence, tadpoles were given a daily diet of green algae and *Daphnia magna*, and after metamorphosis they were switched to a commercially manufactured frog feed (Cargill Feed Co., LTD, Nanjing, China).

### Effects on the tadpoles

In this study, healthy tadpoles at the Nieuwkoop-Faber stage 46/47 with a uniform body weight (~22 mg) and body length (~5 mm) were selected and randomly divided into five treatment groups: those receiving 100 μg L^–1^ purified Cry1Ca (measured value 30.27 ± 2.15 μg L^–1^, n = 8), 10 μg L^–1^ purified Cry1Ca (measured value 3.32 ± 0.12 μg L^–1^, n = 8), T1C-19 rice straw (average Cry1Ca protein concentration 2.15 ± 0.76 μg g^–1^, n = 8), MH63 rice straw, and a blank control group. There were 64 tadpoles in each of the groups given Cry1Ca and in the blank control group. The two groups given rice straw each contained 48 tadpoles. The feeding containers were 1-L beakers, and four tadpoles were raised in each beaker. The rearing water was replaced every 2 days over the experimental period. For the rice straw groups, 0.5 g of T1C-19 or MH63 rice straw was added to each beaker when the rearing water was replaced.

The development and survival of the tadpoles were recorded twice per day (9:00 am, 9:00 pm) until metamorphosis occurred. Dead tadpoles were recorded and an autopsy was immediately conducted. Tadpoles at the near-death stage (indicated by swimming with the belly up, or tumble swimming) were removed, anesthetized with MS-222 and dissected. At the end of the experiment, the tadpoles were anaesthetized by immersion in an ice water mixture containing 0.1% MS-222, and their body weights and body lengths (from the tip of the snout to the tip of the cloaca) were measured. Then, the froglets were dissected, and their hearts, livers, kidneys, fat bodies and intestines were collected and weighed. At the same time, the separated livers were placed in 1.5-mL centrifuge tubes for cryopreservation and used to determine the following parameters: albumin (ALB), total protein (TP), alkaline phosphatase (AKP) activity, alanine aminotransferase (ALT) activity, aspartate aminotransferase (AST) activity and cholinesterase (CHE) activity. The determination methods and the operational processes were completed in strict accordance with the appropriate kits’ directions (JianCheng Bioengineering Institute, Nanjing, China).

### Effects on the froglets

A total of 120 froglets with uniform body weight (~1 g) were equally divided into three experimental groups (T1C-19, MH63 and blank control groups). Each experimental group was randomly divided into four glass jars (20 × 34 × 24 cm) to give four replicates, and each replicate group included 10 froglets. The froglets of the blank control group were fed diets designed for *Rana catesbeiana* (Cargill Feed Co., LTD, Nanjing, China). The diets of the froglets in the T1C-19 and MH63 groups were prepared according to the conventional nutrient composition of the blank control diet, but the experimental rice grain was the largest component. The two self-made test diets contained 30% rice grain, and the detailed diet compositions are shown in Table 1. The conventional nutrient composition of the diets was measured by the Beijing Research Institute for Nutritional Sources (Beijing, China), and the results are shown in Table 2. The Cry1Ca concentrations in the diets were 0.17 ± 0.03 (n = 4), 0 and 0 μg g^–1^ for the T1C-19, MH63 and blank control diets, respectively.

**Table 1:** Composition of the transgenic *cry1Ca* rice (T1C-19) and the non-transformed isoline (MH63) test diets for *Xenopus laevis* froglets.

Ingredient (%)	T1C-19 diet	MH63 diet
Maize	14.50	14.50
Soybean meal	14.50	14.50
Fishmeal	40.00	40.00
T1C-19 rice grain	30.00	–
MH63 rice grain	–	30.00
Additive ^*^	1.00	1.00
Total	100.00	100.00

* Contains in mg kg^–1^ diet: iron, 70; copper, 11; manganese, 70; zinc, 65; iodine, 0.49; selenium, 0.3; vitamin A, 8000 (IU); vitamin D, 2400 (IU); vitamin E, 20 (IU); vitamin K, 0.5 (IU); vitamin B1, 2; vitamin B2, 8; vitamin B6, 3.5; vitamin B12, 0.01; calcium pantothenate, 20; niacin, 35; folic acid, 0.75; and biotin, 0.26.

**Table 2:** Conventional nutrient composition of the transgenic *cry1Ca* rice (T1C-19), the non-transformed isoline (MH63) and the blank control diets for *Xenopus laevis* froglets (n = 1).

Ingredient	T1C-19	MH63	Blank control
Crude protein (%)	38.60	39.30	41.80
Crude fat (g kg^–1^)	59.00	61.00	81.00
Crude fiber (%)	2.90	2.80	2.30
Crude ash (%)	8.20	8.10	9.90
Moisture content (%)	9.40	9.40	6.50
Total phosphorus (%)	1.16	1.16	1.39
Calcium (g kg^–1^)	14.00	13.00	16.00

Note: Data in the table are measured values.

From 0 to 30 days, 31 to 60 days and 61 to 90 days, 2.5, 3.0 and 4.0 g, respectively, of feed was supplied daily and each jar contained 2, 3 and 4 L, respectively, of dechlorinated water. The self-made feed was mixed with water in a 1:1 ratio before each feeding. The prepared feed was pressed into strips using a 5-mL injector, and then the strips were cut into feed portions of ~2 to 4 mm. These prepared portions were placed into containers to feed *X*. *laevis*. The rearing water was monitored daily to maintain 20–22°C and renewed every 3 days. The froglets were weighed and recorded every 15 days (0, 15, 30, 45, 60, 75 and 90 d) for a total of seven weighings during the 90-day experimental period.

After the froglets were fed for 90 days, they were anaesthetized by immersion in ice water mixture containing 0.1% MS-222 and then dissected. The livers and fat bodies were weighed, and the ratios between liver or fat body weight and body weight were calculated. Then, eight froglets (four males and four females) in each group were randomly selected and their stomachs, intestinal tracts, hearts, livers, spleens, testes and ovaries were fixed in 4% neutral formalin for 48 h, embedded in paraffin, sectioned and stained with hematoxylin and eosin. The samples were analyzed and photographed using a microscope (BX63, Olympus, Japan).

### Data analysis

All data are represented as means ± standard error (SE) unless otherwise indicated. The comparisons of the mortality rates between the different treatment groups and the blank control group were conducted using the Chi-square test Bonferroni corrected. Differences in the time for complete metamorphosis (TCM), body weight, body length, organ weight and liver enzyme activities among different treatments were analyzed using a one-way analysis of variance (ANOVA) followed by a least significant difference (LSD) multiple comparison test. Differences were considered significant at *P* < 0.05.

## Results

### Survival and development of the tadpoles

**Fig 1:**
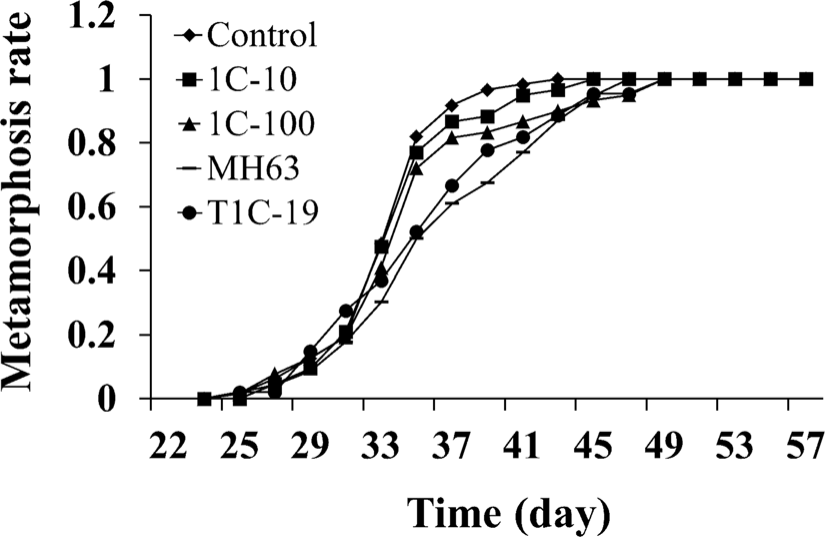
The time to completed metamorphosis (TCM) curves of *Xenopus laevis* tadpoles exposed to purified Cry1Ca and transgenic *cry1Ca* rice (T1C-19) straw. Control: blank control; 1C-10: 10 μg L^–1^ Cry1Ca protein; 1C-100: 100 μg L^–1^ Cry1Ca protein; MH63: MH63 rice straw; T1C-19: T1C-19 rice straw.

The metamorphosis rates on different dates during the different treatments were analyzed and are shown in Fig 1. The order of the TCM for the groups was: MH63 rice straw (35.27 ± 0.93 d) > T1C-19 rice straw (34.29 ± 0.80 d) > 100 μg L^–1^ Cry1Ca protein (33.91 ± 0.58 d) > 10 μg L^–1^ Cry1Ca protein (33.50 ± 0.48 d) > blank control (33.20 ± 0.41 d), but there were no significant differences among the five different treatments (one way ANOVA: *df* = 4, 67; *F* = 1.53; *P* = 0.20).

The effects of different treatments on the tadpoles’ survival rate, body weight and body length are shown in Table 3. The results showed that, the survival rates of rice straw-supplemented groups were lower than those of the blank control group and the Cry1Ca-supplemented groups, but the differences were not significant. Meanwhile, there were no significant differences in the body length among the five treatments (one way ANOVA: *df* = 4, 67; *F* = 1.54; *P* = 0.20). After long-term exposure to 10 and 100 μg L^–1^ Cry1Ca, there were no significant differences in the larval body weights when compared with those in the blank control. The body weight in the MH63 rice straw group was significantly lower than that in the blank control group, but no significant difference was observed between the T1C-19 and MH63 rice straw groups (Table 3).

**Table 3:** Survival and development of *Xenopus laevis* tadpoles exposed to purified Cry1Ca protein, transgenic *cry1Ca* rice (T1C-19) straw and non-transformed isoline (MH63) straw. The experiment was initiated with 64 (n = 16 for 10, 100 μg L^–1^ Cry1Ca protein and blank control groups) or 48 (n = 12 for T1C-19 and MH63 rice straw groups) tadpoles.

Index	Blank control	10 μg L^–1^ Cry1Ca	100 μg L^–1^ Cry1Ca	T1C-19 rice straw	MH63 rice straw	Statistics (One-way ANOVA)
Survival rate (%)^*^	93.75	90.63	89.06	87.50	77.08	–
Body length (mm)	19.83 ± 0.27	20.24 ± 0.16	19.94 ± 0.19	20.10 ± 0.11	19.50 ± 0.28	*df* = 4, 67; *F* = 1.54; *P* = 0.20
Body weight (g)	1.07 ± 0.04^a^	1.03 ± 0.03^a^	0.97 ± 0.03^ab^	0.96 ± 0.03^ab^	0.89 ± 0.03^b^	*df* = 4, 67; *F* = 4.25; *P* < 0.01
Fat body (mg)	10.59 ± 0.90^a^	10.27 ± 0.98^a^	8.18 ± 0.95^a^	5.80 ± 0.89^b^	4.16 ± 0.72^b^	*df* = 4, 67; *F* = 8.20; *P* < 0.01
Heart (mg)	7.27 ± 0.23^ab^	7.56 ± 0.41^a^	6.59 ± 0.34^ab^	7.34 ± 0.50^ab^	6.00 ± 0.25^b^	*df* = 4, 67; *F* = 2.95; *P* = 0.03
Intestine (mg)	32.79 ± 1.49	34.39 ± 1.19	31.70 ± 1.36	31.30 ± 0.81	32.03 ± 1.36	*df* = 4, 67; *F* = 0.90; *P* = 0.47
Kidney (mg)	9.08 ± 0.50	8.36 ± 0.43	8.71 ± 0.45	7.91 ± 0.42	7.98 ± 0.53	*df* = 4, 67; *F* = 1.07; *P* = 0.38
Liver (mg)	37.07 ± 1.05^a^	36.56 ± 1.47^a^	34.42 ± 1.31^a^	27.91 ± 1.34^b^	27.96 ± 2.04^b^	*df* = 4, 67; *F* = 7.81; *P* < 0.01

Data in the table are means ± SE except for the survival rate

* Chi-square test with Bonferroni corrections (adjusted α = 0.0125)

Different small letters within the same row mean significant difference (*P* < 0.05).

### Organ development of the tadpoles

There were no significant differences in the kidney or intestinal weights among the five treatments (all *P* > 0.05, Table 3). When compared with the blank control, the weights of the heart, liver as well as the fat body in the groups supplied 10 and 100 μg L^–1^ Cry1Ca demonstrated no significant differences. The weights of the liver and fat body in the T1C-19 and MH63 rice straw groups were significantly lower than those of the blank control group and the Cry1Ca-supplemented groups. For heart weight, the only difference among the five treatments was observed between the T1C-19 rice straw and 10 μg L^–1^ Cry1Ca-supplemented groups. However, none of the measured indices showed any significant difference between the T1C-19 and MH63 rice straw groups (Table 3).

### Liver enzyme activity of the tadpoles

Among the five treatment groups, no significant differences were found in TP, ALT, AST and AKP (all *P* > 0.05, Table 4). The levels of ALB in the rice straw–supplemented groups were significantly lower than those of the blank control and Cry1Ca-supplemented groups (*P* < 0.05, Table 4). The level of CHE in the MH63 rice straw–supplemented group was significantly lower than those of the blank control and Cry1Ca-supplemented groups. However, no significant differences between the T1C-19 and MH63 rice straw groups were observed (Table 4).

**Table 4:** Protein and enzyme activity in livers of *Xenopus laevis* tadpoles exposed to purified Cry1Ca, transgenic *cry1Ca* rice (T1C-19) straw and non-transformed isoline (MH63) straw (n = 24).

Index	Blank control	10 μg L^–1^ Cry1Ca	100 μg L^–1^ Cry1Ca	T1C-19 rice straw	MH63 rice straw	Statistics (One-way ANOVA)
ALB (g g^–1^)	0.15 ± 0.01^a^	0.14 ± 0.01^a^	0.14 ± 0.01^a^	0.11 ± 0.01^b^	0.11 ± 0.00^b^	*df* = 4, 115; *F* = 13.02; *P* < 0.01
TP (g g^–1^)	0.21 ± 0.01	0.20 ± 0.01	0.21 ± 0.01	0.19 ± 0.01	0.19 ± 0.01	*df* = 4, 115; *F* = 1.41; *P* = 0.24
AKP (U gprot^–1^)	20.30 ± 1.50	19.42 ± 1.29	20.06 ± 1.75	17.67 ± 1.22	19.35 ± 1.12	*df* = 4, 115; *F* = 0.54; *P* = 0.70
ALT (U gprot^–1^)	14.49 ± 0.70	15.38 ± 0.79	14.65 ± 0.75	16.40 ± 0.99	15.86 ± 0.91	*df* = 4, 115; *F* = 0.93; *P* = 0.45
AST (U gprot^–1^)	17.66 ± 1.21	18.71 ± 0.93	16.75 ± 1.00	18.68 ± 1.34	17.15 ± 0.97	*df* = 4, 115; *F* = 0.65; *P* = 0.63
CHE (U mgprot^–1^)	6.43 ± 0.40^ab^	7.05 ± 0.32^a^	6.68 ± 0.42^ab^	5.73 ± 0.41^bc^	5.19 ± 0.30^c^	*df* = 4, 115; *F* = 4.00; *P* < 0.01

Data in the table are means ± SE

Different small letters within the same row mean significant difference (*P* < 0.05).

### Survival and development of froglets

**Fig 2:**
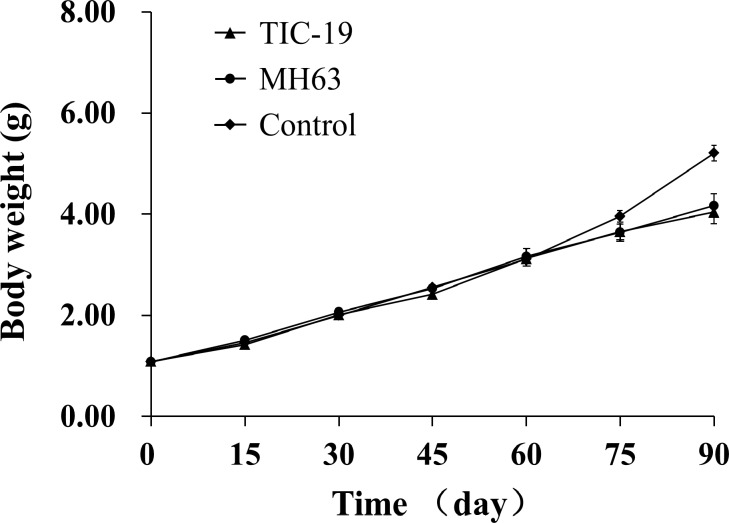
The growth curves of *Xenopus laevis* froglets (body weight) when fed diets containing transgenic *cry1Ca* rice (T1C-19) or non-transformed rice isoline (MH63) grains, or a blank control diet for 90 days (n = 4).

The growth curves of froglets fed control, MH63 rice or T1C-19 rice diets are shown in Fig 2. The fitted curve for the blank control is y = 0.6266 x + 0.2160 (r^2^ = 0.9789), for the MH63 dietary group is y = 0.5060 x + 0.5490 (r^2^ = 0.9971) and for the T1C-19 dietary group is y = 0.5024 x + 0.5100 (r^2^ = 0.9908). The data suggested that the growth rate of the blank control group was greater than those of the MH63 and T1C-19 dietary groups, which had almost overlapping growth curves (Fig 2).

During the feeding experiment, there were no deaths in the three treatment groups, so the survival rate was 100% (Table 5). After 90 days of feeding, the mean final body weight, daily weight gain, liver weight, fat body weight and fat body/body weight ratio of the froglets in the blank control group were significantly higher than those of the T1C-19 and MH63 dietary groups (all *P* < 0.05, Table 5), whereas no significant differences were observed between the T1C-19 and MH63 groups (Table 5). No significant difference in the hepatosomatic index was observed between any group.

**Table 5:** Survival and organ weight of *Xenopus laevis* froglets fed diets containing transgenic *cry1Ca* rice (T1C-19) grains and non-transformed isoline (MH63) grains for 90 days (n = 4).

Index	Blank control	T1C-19	MH63	Statistic (One-way ANOVA)
Survival rate (%)	100	100	100	–
Initial weight (g)	1.08 ± 0.03	1.09 ± 0.03	1.08 ± 0.03	*df* = 2, 9; *F* = 0.02; *P* = 0.98
Final weight (g)	5.21 ± 0.15^b^	4.04 ± 0.23^a^	4.18 ± 0.23^a^	*df* = 2, 9; *F* = 10.21; *P* < 0.01
Daily weight gain (g)*	0.05 ± 0.00^b^	0.03 ± 0.00^a^	0.03 ± 0.00^a^	*df* = 2, 9; *F* = 46.48; *P* < 0.01
Liver weight (g)	0.21 ± 0.01^b^	0.15 ± 0.01^a^	0.16 ± 0.01^a^	*df* = 2, 9; *F* = 11.51; *P* < 0.01
Fat body (g)	0.16 ± 0.01^b^	0.11 ± 0.01^a^	0.12 ± 0.01^a^	*df* = 2, 9; *F* = 16.45; *P* < 0.01
Liver/body weight (%)	3.96 ± 0.07	3.76 ± 0.08	3.83 ± 0.09	*df* = 2, 9; *F* = 1.69; *P* = 0.19
Fat body/body weight (%)	3.15 ± 0.07^b^	2.79 ± 0.08^a^	2.79 ± 0.08^a^	*df* = 2, 9; *F* = 7.32; *P* < 0.01

Data in the table are means ± SE except for the survival rate

* Daily weight gain = (Final weight–initial weight) / 90 days

Different small letters within the same row mean significant difference (*P* < 0.05).

### Gross necropsy and histopathology of froglets

**Fig 3:**
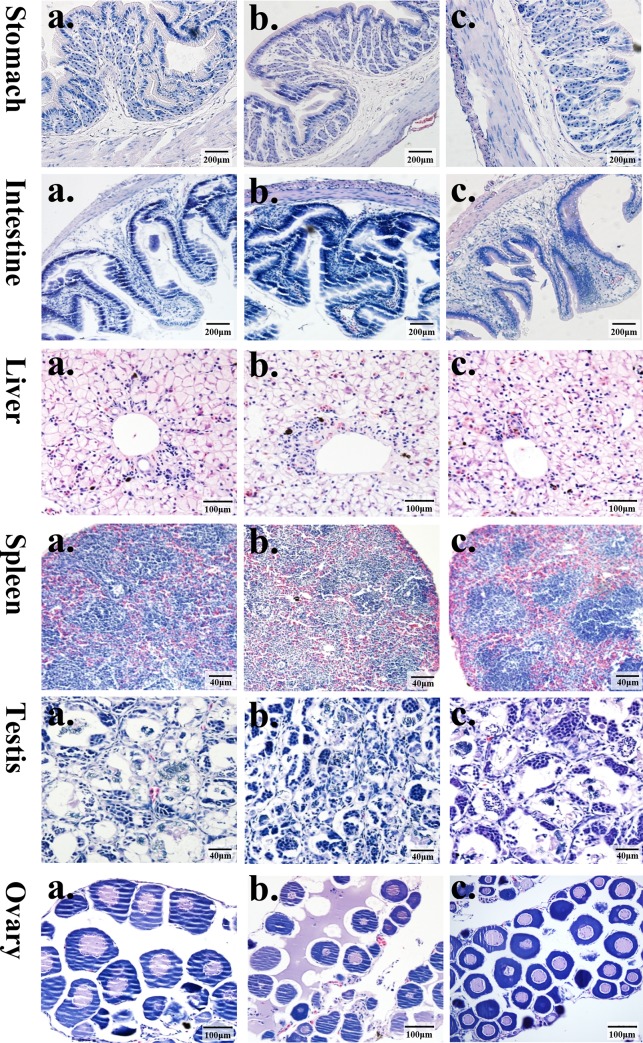
Histopathological staining of tissues from *Xenopus laevis* froglets after consuming the transgenic *cry1Ca* rice (T1C-19) test diet (a), the non-transformed isoline (MH63) test diet (b), or the blank control diet (c) for 90 days.

Histological examinations of the stomach, intestine (ileum), liver, spleen, testes and ovaries are shown in Fig 3. There were no gross pathological findings during the necropsies, and no group-related histopathological abnormalities were observed.

## Discussion

Until now, studies on the effects of toxic and harmful substances on the development of *X*. *laevis* tadpoles have focused on insecticides, such as endosulfan and triazophos [30,31], herbicides, such as acetochlor and butachlor ammonium [32], and heavy metal ions found in industrial wastewater, such as Cu^2+^ and Zn^2+^ [33]. However, there have been no reports on the effects of *Bt* crops and their expression products on tadpole development. Our previous study showed that the peak water concentration of Cry1Ca protein leached from T1C-19 straw under laboratory conditions was 5.18 μg L^–1^ in a worst-case scenario and the maximum value was 6.51 μg L^–1^ [34]. Moreover, under natural conditions, the Bt content in the water is less than 100 ng L^–1^ [13,20]. In this study, although the concentrations of Bt protein added were low (10 and 100 μg L^–1^), they were ~2- to 20-fold higher than that in the worst-case scenario [9] or in the natural environment, which met the requirements of a Tier-1 test system [35] for the environmental safety assessment of *Bt* crops.

Oka et al. studied the effects of atrazine on *X*. *laevis* tadpoles and found that 35–56 days were needed for the 46-stage tadpoles to complete their metamorphoses [36]. In contrast, Coadya et al. showed that 72.8 ± 0.4 days were needed for the tadpoles in all experimental groups to complete their metamorphoses [37]. In the present study, tadpoles in the blank control group needed ~33 days from the 46-stage to complete metamorphosis. The differences in the TCMs could be caused by many factors, such as hazard substances, water quality, nutrient supply or feeding density [38,39]. Our results showed that the TCMs of the five different treatments were relatively consistent, indicating that the effects of pure Cry1Ca protein or rice straw on the TCM were not significant. However, most of the detection indices, such as liver and fat body weights and liver ALB level, in the rice straw groups showed significant differences when compared with those of the blank control group. Possible reasons may include a change in water quality, such as the pH decreasing to ~4.5 [34], which is lower than the optimal pH value (6 to 8) for tadpole growth, after the addition of the rice straw. In addition, allelochemicals, such as phenolic acids, hydroxamic acids, fatty acids, terpenes, indole and phenolic acids [40], were probably released from the rice straw, and these substances may have had adverse effects on tadpole growth. However, the differences were not significant between the T1C-19 and MH63 rice straw groups, which indicated that the insertion of the *Bt* gene did not produce any significant unintended effects on tadpole development.

With regards to the impact of *Bt* crops on non-target organisms, studies have shown that there were no significant changes in some growth indices, but some physiological and biochemical indices were significantly changed [41]. However, in this study, the examined indices of the groups supplied 100 μg L^–1^ and 10 μg L^–1^ pure Cry1Ca protein showed no significant differences when compared with those of the blank control group. There were significant differences between the ALB and CHE levels observed in the T1C-19 and MH63 rice straw groups and the blank control group, but the differences were not significant between the T1C-19 and MH63 groups. The other indices showed no significant differences with the blank control group. These results were consistent with the growth and development detection indices of the present experiment. They also confirmed the hypothesis that the Cry1Ca protein and the T1C-19 rice did not have negative impacts on tadpole growth.

Cry proteins from Bt have a high specificity, acting via specific receptors on the intestinal wall of the epithelial cells of sensitive insects, causing paralysis of the insect’s targeted intestinal cells, which affects their food consumption [42]. The intestinal epithelial cells of other animals do not have the protein’s binding site, and thus exposure to the protein should not affect other animals. At present, food and feed safety research on *Bt* rice mainly uses rats [43,44], broiler chickens [45] and pigs [46] as research subjects for 30- or 90-day toxicity tests or allergenicity tests, and all of these studies showed that the safety of the Bt-transgenic and non-transformed parental lines were comparable.

Cry1C is a Bt protein that shows good resistance to lepidopteran pests [47], as well as good heat stability. However, artificial gastrointestinal fluids can quickly digest this protein *in vitro*, and no adverse effects were observed in rats fed 5 g kg^–1^ protein per weight [48]. In the present study, to assess the safety of Bt rice on a frog species, we exposed *X*. *laevis* froglets to Cry1Ca proteins by feeding them a diet containing transgenic *cry1Ca* rice grains for 90 days. There were no significant differences in the survival rates between the three different treatments, whereas the body, liver and fat body weights in the blank control group were significantly higher than those in the test T1C-19 and MH63 dietary groups. However, there was no significant difference between the rice dietary groups. The differences between the rice fed groups and the blank control may have been caused by a slight difference between the conventional nutrient composition of the blank control and that of the self-made test diets (Table 2). Additionally, the blank control was an aquacultureal feed, whereas the feed of the test groups was self-made and dissolved in water for a short time, and thus it may not have been conducive to froglet uptake. In our previous study, there were no significant differences among the diets containing rice grain and blank control groups in terms of body weight and organ weights [26], this may due to different feed formulation, and different feed production methods compared with the present study. However, there were no significant poisoning symptoms in the test animals and no abnormal pathology was observed by gross anatomy after 90 days of feeding in the present study. In addition, there were no abnormal phenomena in the microstructures of the stomachs, intestinal tracts, livers, kidneys or other important organs (Fig 3).

## Conclusions

In the present study, *X*. *laevis* were exposed to Bt proteins by adding high doses of purified Cry1Ca protein or T1C-19 rice straw to the rearing water, or by feeding them a diet containing T1C-19 rice grains, which carry the gene encoding Cry1Ca. However, the development of tadpoles and froglets was not adversely affected. Based on these results, we conclude that the planting of transgenic *cry1Ca* rice will not adversely affect frog development.

## References

[1] Lu C (2010) The first approved transgenic rice in China. GM crops 1(3): 113–115. 10.4161/gmcr.1.3.12377 21865866

[2] Akhtar ZR, Tian JC, Chen Y, Fang Q, Hu C, Chen M, et al (2010) Impacts of six bt rice lines on nontarget rice feeding thrips under laboratory and field conditions. Environmental Entomology 39: 715–726. 10.1603/EN09095 20388307

[3] Mannakkara A, Niu L, Ma W, Lei C (2013) Zero effect of Bt rice on expression of genes coding for digestion, detoxification and immune responses and developmental performances of Brown Planthopper *Nilaparvata lugens* (Stål). Journal of Insect Physiology 59: 985–993. 10.1016/j.jinsphys.2013.07.009 23920284

[4] Tian JC, Chen Y, Li ZL, Li K, Chen M, Peng YF, et al (2012) Transgenic Cry1Ab rice does not impact ecological fitness and predation of a generalist spider. PLoS ONE 7(4): e35164 10.1371/journal.pone.0035164 22511982 PMC3325204

[5] Wang YY, Li YH, Romeis J, Chen XP, Zhang J, Chen HY, et al (2012) Consumption of *Bt* rice pollen expressing Cry2Aa does not cause adverse effects on adult *Chrysoperla sinica* Tjeder (Neuroptera: Chrysopidae). Biological Control 61: 246–251.

[6] Yao HW, Jiang CY, Ye GY, Hu C, Peng YF (2008) Toxicological assessment of pollen from different Bt rice lines on *Bombyx mori* (Lepidoptera: Bombyxidae). Environmental Entomology 37: 825–837. 18559190 10.1603/0046-225x(2008)37[825:taopfd]2.0.co;2

[7] Yang Y, Liu Y, Cao F, Chen X, Cheng L, Romeis J, et al (2014) Consumption of Bt rice pollen containing Cry1C or Cry2A protein poses a low to negligible risk to the silkworm *Bombyx mori* (Lepidoptera: Bombyxidae). PLoS ONE 9(7): e102302 10.1371/journal.pone.0102302 25014054 PMC4094503

[8] Liu W, Lu HH, Wu WX, Wei QK, Chen YX, Thies JE (2008) Transgenic Bt rice does not affect enzyme activities and microbial composition in the rhizosphere during crop development. Soil Biology & Biochemistry 40: 475–486.

[9] Carstens K, Anderson J, Bachman P, Schrijver AD, Dively G, Federici B, et al (2012) Genetically modified crops and aquatic ecosystems: considerations for environmental risk assessment and nontarget organism testing. Transgenic Research 21: 813–842. 10.1007/s11248-011-9569-8 22120952 PMC3394238

[10] Viktorov AG (2011) Transfer of Bt corn byproducts from terrestrial to stream ecosystems. Russian Journal of Plant Physiology 58: 543–548.

[11] Douville M, Gagné F, Blaise C, André C (2007) Occurrence and persistence of *Bacillus thuringiensis* (Bt) and transgenic Bt corn *cry1Ab* gene from an aquatic environment. Ecotoxicology and Environmental Safety 66: 195–203. 16499967 10.1016/j.ecoenv.2006.01.002

[12] Douville M, Gagné F, André C, Blaise C (2009) Occurrence of the transgenic Bt corn *cry1Ab* gene in freshwater mussle (*Elliptio complanata*) near corn field: Evidence of exposure by bacterial ingestion. Ecotoxicology and Environmental Safety 72: 17–25. 10.1016/j.ecoenv.2008.02.006 18397807

[13] Tank JL, Rosi-Marshall EJ, Royer TV, Whiles MR, Griffiths NA, Frauendorf TC, et al (2010) Occurrence of maize detritus and a transgenic insecticidal protein (Cry1Ab) within the stream network of an agricultural landscape. Proceedings of the National Academy of Sciences of the United States of America 107: 17645–17650. 10.1073/pnas.1006925107 20876106 PMC2955116

[14] Bøhn T, Primicerio R, Hessen DO, Traavik T (2008) Reduced fitness of *Daphnia magna* fed a Bt-transgenic maize variety. Archives of Environmental Contamination and Toxicology 55: 584–592. 10.1007/s00244-008-9150-5 18347840

[15] Bøhn T, Traavik T, Primicerio R (2010) Demographic responses of *Daphnia magna* fed transgenic Bt-maize. Ecotoxicology 19: 419–430. 10.1007/s10646-009-0427-x 19859805 PMC2811247

[16] Raybould A, Vlachos D (2011) Non-target organism effects tests on Vip3A and their application to the ecological risk assessment for cultivation of MIR162 maize. Transgenic Research 20: 599–611. 10.1007/s11248-010-9442-1 20839052

[17] Rosi-Marshall EJ, Tank JL, Royer TV, Whiles MR, Evans-White M, Chambers C, et al (2007) Toxins in transgenic crop byproducts may affect headwater stream ecosystems. Proceedings of the National Academy of Sciences of the United States of America 104, 16204–16208. 17923672 10.1073/pnas.0707177104 PMC2042185

[18] Chambers CP, Whiles MR, Rosi-Marshall EJ, Tank JL, Royer TV, Griffiths NA, et al (2010) Responses of stream macroinvertebrates to Bt maize leaf detritus. Ecological Applications 20: 1949–1960. 21049882 10.1890/09-0598.1

[19] Jensen PD, Dively GP, Swan CM, Lamp WO (2010). Exposure and nontarget effects of transgenic Bt corn debris in streams. Environmental Entomology 39: 707–714. 10.1603/EN09037 20388306

[20] Wang YM, Hu HW, Huang JC, Li JH, Liu B, Zhang GA (2013) Determination of the movement and persistence of Cry1Ab/1Ac protein released from Bt transgenic rice under field and hydroponic conditions. Soil Biology & Biochemistry 58: 107–114.

[21] Hayes TB, Case P, Chui S, Chung D, Haeffele C, Haston K, et al (2006) Pesticide mixtures, endocrine disruption, and amphibian declines: are we underestimating the impact? Environmental Health Perspectives 114: 40–50. 10.1289/ehp.8051 PMC1874187 16818245

[22] Blaustein AR, Han BA, Relyea RA, Johnson PTJ, Buck JC, Gervasi SS, et al (2011) The complexity of amphibian population declines: understanding the role of cofactors in driving amphibian losses. Annals of the New York Academy of Sciences 1223: 108–119. 10.1111/j.1749-6632.2010.05909.x 21449968

[23] Zhang QL, Li YH, Hua HX, Yang CJ, Wu HJ, Peng YF (2013) Exposure degree of important non-target arthropods to Cry2Aa in Bt rice fields. Chinese Journal of Application Ecology 24: 1647–1651. 24066553

[24] Lu YH, Wu KM, Jiang YY, Xia B, Li P, Feng HQ, et al (2010) Mirid bug outbreaks in multiple crops correlated with wide-scale adoption of Bt cotton in China. Science 328: 1151–1154. 10.1126/science.1187881 20466880

[25] O'Rourke DP (2007) Amphibians used in research and teaching. ILAR Journal 48: 183–187. 17592182 10.1093/ilar.48.3.183

[26] Zhu HJ, Chen Y, Li YH, Wang JM, Ding JT, Chen XP, et al (2015) A 90-day safety assessment of genetically modified rice expressing Cry1Ab/1Ac protein using an aquatic animal model. Journal of Agriculture and Food Chemistry 63(14): 3627–3633. 10.1021/jf5055547 25822065

[27] Zheng XS, Yang YJ, Xu HX, Chen H, Wang BJ, Lin YJ, et al (2011) Resistance performances of transgenic bt rice lines T_2A_-1 and T1c-19 against *Cnaphalocrocis medinalis* (Lepidoptera: Pyralidae). Journal of Economic Entomology 104: 1730–1735. 22066204 10.1603/ec10389

[28] Wang YN, Ke KQ, Li YH, Han LZ, Liu YM, Hua HX, et al (2014) Comparison of three transgenic Bt rice lines for insecticidal protein expression and resistance against a target pest, *Chilo suppressalis* (Lepidoptera: Crambidae). Insect Science 10.1111/1744-7917.12178 25284137

[29] Li Y, Chen X, Hu L, Romeis J, Peng Y (2014) Bt rice producing Cry1C protein does not have direct detrimental effects on the green lacewing *Chrysoperla sinica* (Tjeder). Environmental Toxicology and Chemistry 33: 1391–1397. 10.1002/etc.2567 24619941

[30] Jones DK, Hammond JI, Relyea RA (2009) Very highly toxic effects of endosulfan across nine speices of tadpoles: lag effects and family-level sensitivity. Environmental Toxicology and Chemistry 28: 1939–1945. 10.1897/09-033.1 19358624

[31] Sparling DW, Fellers G (2007) Comparative toxicity of chlorpyrifos, diazinon, malathion and their oxon derivatives to larval *Rana boylii* . Environment Pollution 147: 535–539. 10.1016/j.envpol.2006.10.036 17218044

[32] Geng BR, Yao D, Xue QQ (2005) Acute toxicity of the pesticide dichlorvos and the herbicide butachlor to tadpoles of four anuran species. Bulletin of Environmental Contamination and Toxicology 75: 343–349. 16222508 10.1007/s00128-005-0759-z

[33] Dobrovoljc K, Falnoga I, Žnidarič MT, Mazej D, Ščančar J, Bulog B (2012) Cd, Cu, Zn, Se, and metallothioneins in two amphibians, *Necturus maculosus* (Amphibia, Caudata) and *Bufo bufo* (Amphibia, Anura). Biological Trace Element Research 150: 178–194. 10.1007/s12011-012-9461-2 22700180

[34] Wang J, Chen X, Li Y, Zhu H, Ding J, Peng Y (2014) Effect of straw leachates from Cry1Ca-expressing transgenic rice on the growth of *Chorella Pyrenoidosa* . Environmental Toxicology and Chemistry 33: 1156–1162. 10.1002/etc.2535 24478192

[35] Li YH, Romeis J, Wu KM, Peng YF (2014) Tier-1 assays for assessing the toxicity of insecticidal proteins produced by genetically engineered plants to non-target arthropods. Insect Science 21: 125–134. 10.1111/1744-7917.12044 23956068

[36] Oka T, Tooi O, Mitsui N, Miyahara M, Ohnishi Y, Takase M, et al (2008) Effect of atrazine on metamorphosis and sexual differentiation in *Xenopus laevis* . Aquatic Toxicology 87: 215–226. 10.1016/j.aquatox.2008.02.009 18395276

[37] Coadya KK, Murphy MB, Villeneuve DL, Hecker M, Jones PD, Carr JA, et al (2005) Effects of atrazine on metamorphosis, growth, laryngeal and gonadal development, aromatase activity, and sex steroid concentrations in *Xenopus laevis* . Ecotoxicology and Environmental Safety 62: 160–173. 16112017 10.1016/j.ecoenv.2004.10.010

[38] Indermaur L, Schmidt BR, Tockner K, Schaub M (2010) Spatial variation in abiotic and biotic factors in a floodplain determine anuran body size and growth rate at metamorphosis. Oecologia 163: 637–649. 10.1007/s00442-010-1586-4 20204410

[39] Morey S, Reznick D (2001) Effects of larval density on postmetamorphic spadefoot toads (Spea hammondii). Ecology 82: 510–522.

[40] Rimando AM, Duke SO (2003) Studies on rice allelochemicals In: Smith C W, Dilday R H (Eds). Rice, origin, histroy, technology and production. John Wiley & Sons, Inc., Hoboken, New Jersey, pp. 221–244.

[41] Zhou J, Xiao K, Wei B, Wang Z, Tian Y, Tian Y, et al (2014) Bioaccumulation of Cry1Ab protein from an herbivore reduces anti-oxidant enzyme activities in two spider species. PLoS ONE 9(1): e84724 10.1371/journal.pone.0084724 24454741 PMC3890278

[42] Hofmann C, Vanderbruggen H, Höfte H, Van Rie J, Jansens S, Van Mellaert H (1988) Specificity of *Bacillus thuringiensis* delta-endotoxins is correlated with the presence of high-affinity binding sites in the brush border membrane of target insect midgets. Proceedings of the National Academy of Sciences of the United States of America 85: 7844–7848. 2856194 10.1073/pnas.85.21.7844 PMC282293

[43] Wang ZH, Wang Y, Cui HR, Xia YW, Altosaar I, Shu QY (2002) Toxicological evaluation of transgenic rice flour with a synthetic *cry1Ab* gene from *Bacillus thuringiensis* . Journal of the Science of Food and Agriculture 82: 738–744.

[44] Schrøer M, Poulsen M, Wilcks A, Kroghsbo S, Miller A, Frenzel T, et al (2007) A 90-day safety study of genetically modified rice expressing Cry1Ab protein (*Bacillus thuringiensis* toxin) in Wistar rats. Food and Chemical Toxicology 45: 339–349. 17050059 10.1016/j.fct.2006.09.001

[45] Qin HF (2012) Safety assessment of rice genetically modified with Cry1Ac and sck by feeding studies on broilers. Dissertation, Chinese Academy of Agriculture Science.

[46] Cao ZH, Wang ZB, Gu XH (2014) Residues and organ damage of exogenous gene and protein from transgenic Bt brown rice in growing pigs. Chinese Journal of Animal Nutrition 26: 1908–1915.

[47] Avisar D, Eilenberg H, Keller M, Reznik N, Segal M, Sneh B, et al (2009) The *Bacillus thuringiensis* delta-endotoxin Cry1C as a potential bioinsecticide in plants. Plant Science 176: 315–324.

[48] Cao SS, He XY, Xu WT, Ran WJ, Liang LX, Luo YB, et al (2010) Safety assessment of Cry1C protein from genetically modified rice according to the national standards of PR China for a new food resource. Regulatory Toxicology and Pharmacology 58(3): 474–481. 10.1016/j.yrtph.2010.08.018 20801181

